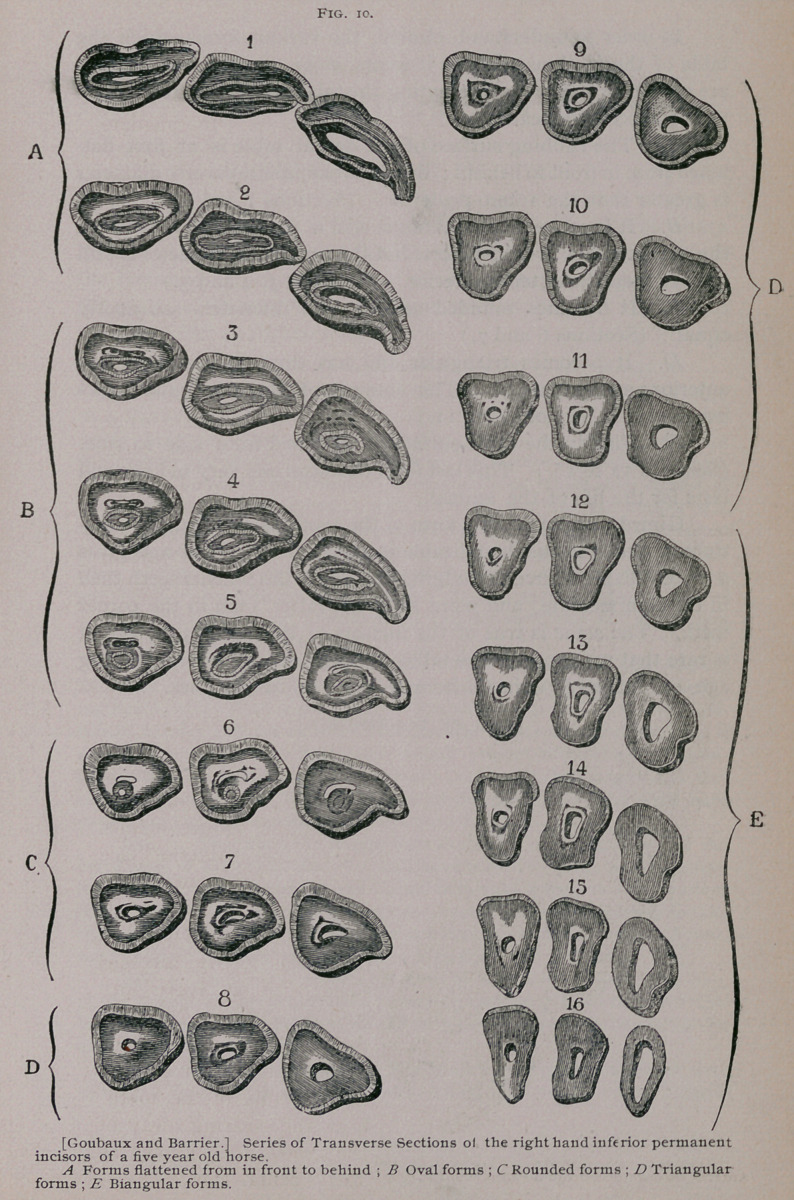# Age of the Horse, Ox, Dog, and Other Domestic Animals

**Published:** 1890-02

**Authors:** R. S. Huidekoper

**Affiliations:** Veterinarian


### AGE OF THE HORSE, OX, DOG, AND OTHER DOMESTICATED ANIMALS.

By R. S. Huidekoper, M.D., Veterinarian.

[*Continued from page 40.*]

*b. Permanent Incisors.*

The permanent incisors are six in number, in each jaw, like the deciduous incisors which they replace; they, like the latter, are known as the pincher teeth, intermediate teeth and corner teeth. They are longer than the milk teeth, are more of a wedge shape, have no constriction or neck separating the crown from the root, and are of a bluish-white, instead of the cream color of the others. The permanent incisors (Fig. 7) are shaped somewhat like an

Fig 7.

[Goubaux and Barrier.] A Virgin Inferior Incisor. *A* Anterior face; *B* Posterior face; *C* Profile.

irregular cone, of which the base corresponds to the crown or free extremity, and the summit to the root or the imbedded extremity. They are curved on their long axis with their convex face in front and their concave face behind, and they are also somewhat twisted on their long axis; the pincher teeth slightly so, the intermediate teeth more so, and the corner teeth sometimes making almost a decided **S**. The free portion, or crown, is flattened from in front to behind; at the level of the gums the two axes have about the same diameter, and the root or embedded portion is flattened from side to side.

The anterior face, which is widest above, is flat from side to side and convex from above to below. It frequently is grooved by a little canal, which is most distinct on the free portion of the tooth. The incisors of the upper jaw are more convex than those of the lower jaw, and they frequently have two little longitudinal canals instead of one. The posterior face is convex and rounded from side to side and concave from above to below. The internal border of the tooth is thicker than the external; this is more marked in the imbedded portion than in the free portion. The free portion, or crown, is flattened from front to behind and is hollowed out by a cavity known as the external cavity or *cup* (Fig. 7, *B* and *C*). In the virgin tooth there is a border in front and a border behind; the latter is not so high as the first, but in old teeth these become worn off until perfectly level, when the extremity of the tooth takes the name of the table of the tooth. (Fig. 8, *a*.)

The external dental cavity *a*, surrounded by the cup of enamel, has an irregular conical form, the base toward the base of the tooth itself and the point imbedded in the tooth but inclined slightly toward the posterior border, especially in the inferior teeth.

This is sometimes an open cup, but it is frequently filled with cement, making the free extremity, even in the virgin tooth, sometimes perfectly solid. The cups in the inferior teeth are less deep than those in the superior incisors.

In the incisors of the lower jaw the pincher teeth

FIG. 8.

[Goubaux and Barrier.] Longitudinal and Antero-posterior Sections of permanent Incisors. *P* Pinchers; *I* Intermediate; *C* Corner.

FIG. 9.

[Goubaux and Barrier.] Longitudinal and Antero-posterior Section of a Permanent Incisor.

have a cup with an average depth of 16 to 18 mm., the cup of intermediate teeth is 18 mm. to 20 mm., and that of the corner teeth is 11 mm. to 13 mm. In the upper jaw the cup of the pinchers is 25 mm. to 27 mm. deep, that of the intermediate teeth 27 mm. to 28 mm. deep, and that of the corner teeth somewhat less, 18 mm. to 20 mm. (Fig. 8, *aaa*). The cups in the lower teeth incline nearer the posterior border of the tooth than in the upper ones.

In the imbedded portion of the tooth there is a large canal which holds the dental pulp or papilla, which is known as the internal dental cavity. (Fig. 8, *b*. Fig. 9, *P*.) Examine Fig. 9 and we find that this cavity occupies the centre of the tooth, in the root, but toward the crown inclines toward the anterior face and penetrates between the latter and the cup of enamel. As the tooth becomes older this cavity becomes filled with an ivory substance, softer and darker colored than the rest of the tooth, and when it appears on the table of the tooth, from the wearing away of the crown it is known as the "dental star."

FIG. 10.

[Goubaux and Barrier.] Series of Transverse Sections of the right hand inferior permanent incisors of a five year old horse.

*A* Forms flattened from in front to behind ; *B* Oval forms ; *C* Rounded forms ; *D* Triangular forms ; *E* Biangular forms.

In order to understand, clearly, the various forms which the table of the tooth assumes as it wears away, a series of sections are shown in Fig. 10, representing the table as it appears from the use of successive years.

*A.* The rubbing surface of the dental table is at first flattened from in front to behind ; that is to say, its transverse diameter is greater than the antero-posterior. (Sections 1 and 2.)

*B.* It becomes oval ; there is still a disproportion between the extent of its two diameters, but the transverse diameter is still greater than the antero-posterior. (Sections 3, 4 and 5.)

*C.* It becomes rounded and its two diameters are nearly equal. (Sections 6 and 7.)

*D.* It becomes triangular and has three (3) borders, one anterior and two lateral. The summit of the triangular looks backward. (Sections 8 to 11.)

*E.* Finally the table surface is flattened from side to side. (Sections 12 to 16.) This last form characterizes very old age and lasts for the life of the animal.

Girard designated this form of the tooth as bi-angular. Distinctive shape in the dental table is much more regular and gives a more reliable source of judging the age in the pincher teeth than in the intermediate, and more so in the latter than in the corner teeth. The same is true of the incisors of the upper jaw, but it is rare that we examine the table of these as an aid in judging age, except in old animals from 16 years to 20 and over.

[TO BE CONTINUED.]

## Figures and Tables

**Fig. 7. f1:**
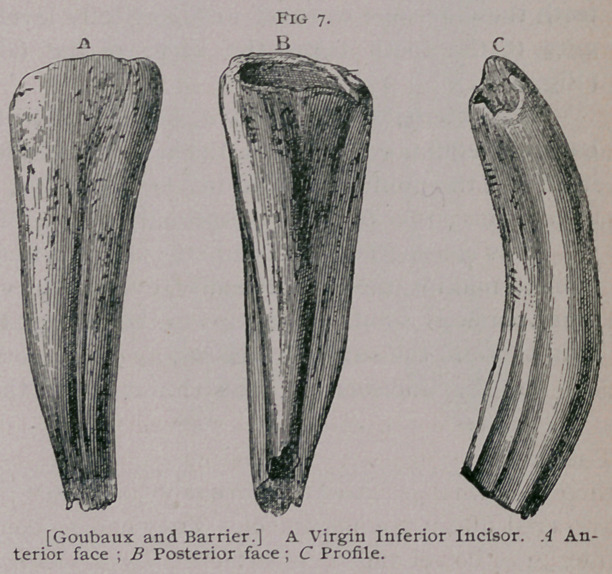


**Fig. 8. f2:**
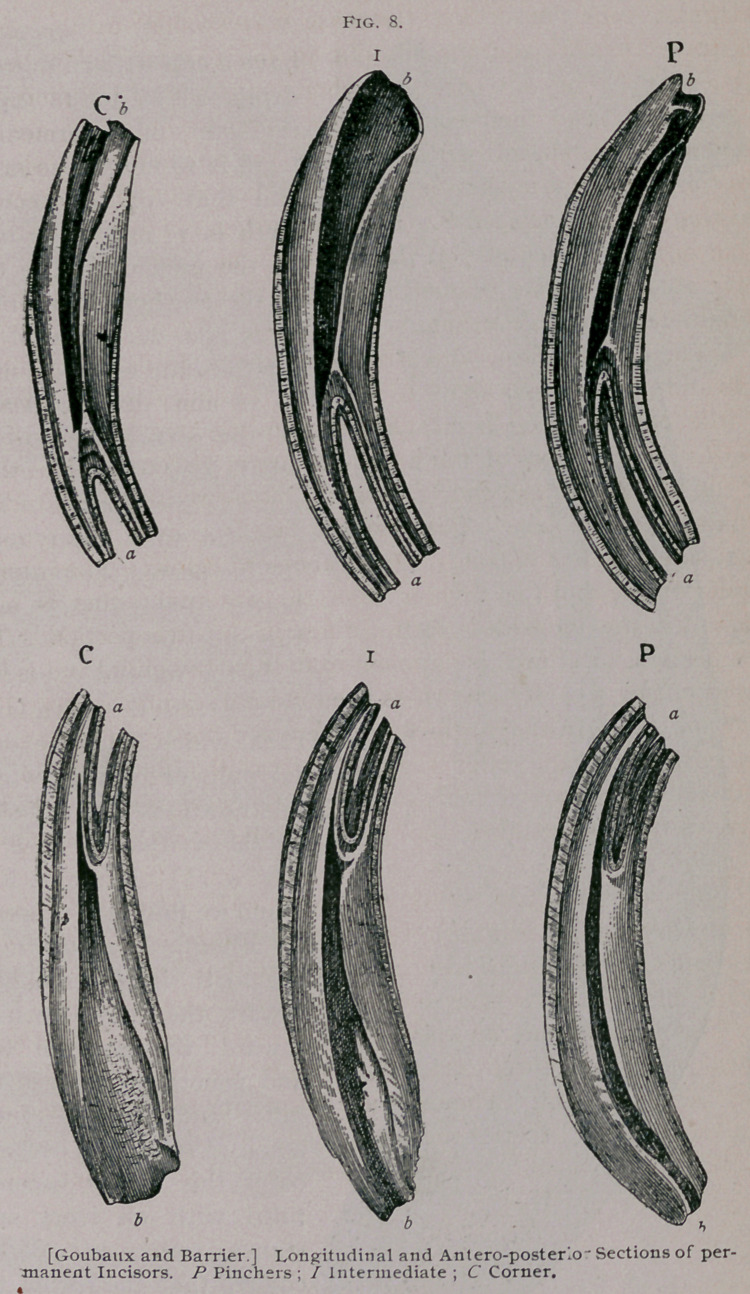


**Fig. 9. f3:**
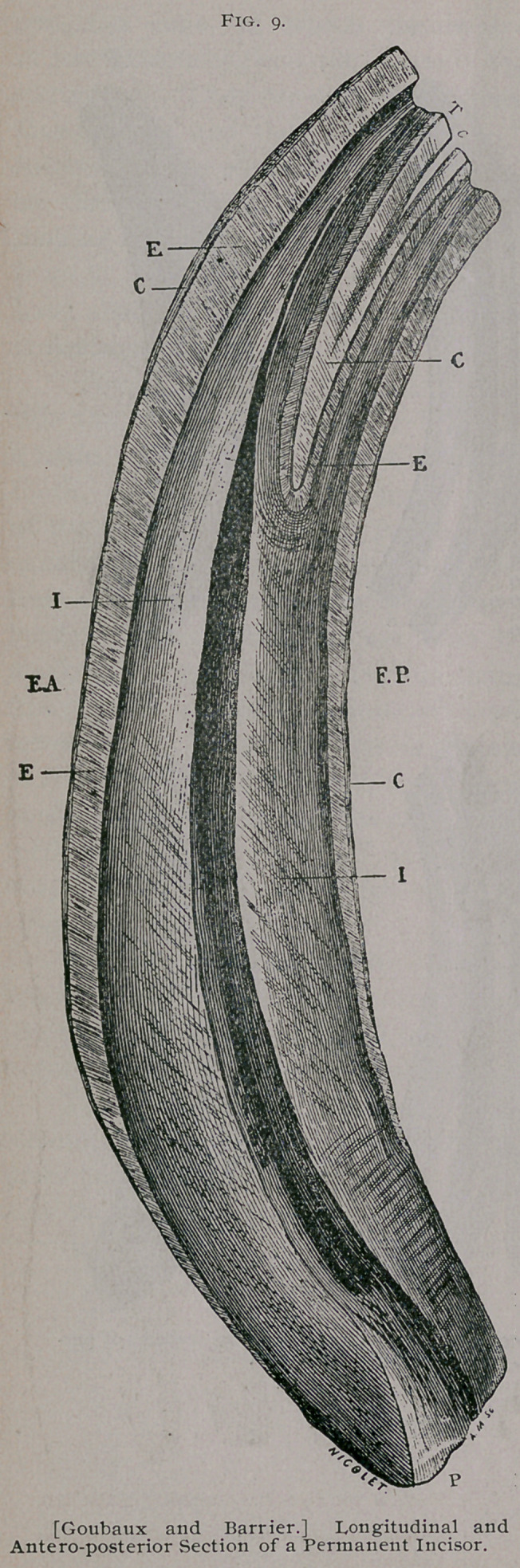


**Fig. 10. f4:**